# Influence of biochar and nitrogen on fine root morphology, physiology, and chemistry of *Acer mono*

**DOI:** 10.1038/s41598-017-05721-2

**Published:** 2017-07-14

**Authors:** Muhammad Razaq, Salahuddin, Hai-long Shen, Hassan Sher, Peng Zhang

**Affiliations:** 1 School of Forestry, Northeast Forestry University, Harbin, China; 2 Agricultural Research Institute, Mingora, Pakistan; 3 Centre for Plants Sciences and Biodiversity University of Swat, Udigram, Pakistan

## Abstract

Fine roots play an important role in the overall functions of individual plants. Previous studies showed that fertilization and available soil resources have a notably profound effect on fine root, but there is lack of study centered on how fine root morphology, physiology, and chemistry respond to biochar with N additions. Different levels of biochar (0, 10, 15, and 20 g) and N (0, 2, 4 and 6 g) were applied to Acer mono seedling plants in a field nursery. The root system morphology and root chemistry and physiology were evaluated in line with root length, root diameter, SRL, N and N: C and root respiration. Biochar and N significantly affected root morphology, chemistry and root respiration. Morphological, chemical and physiological parameters were found to be at their maximum with 20 g biochar and 6 g N; however, no significant effect was noted on fourth- and fifth-order roots. Furthermore, a significant increase in root respiration was recognized with the increase in root tissue N concentration and the negative relationship of root respiration with higher branch order. Thus, overall, study parameters indicate that biochar and nitrogen positively influence the *Acer mono* fine root, and therefore should be used to improve fine root health.

## Introduction

Nursery practices such as sowing, seedbed density, pruning, and fertilisation are usually standardised for individual plant species in order to produce high-quality seedlings^1^. Different fertilisers and nutrients are widely used to improve plant vigour and productivity^2^. Fine roots play a vital role in root systems since they show high physiological activity during nutrient and water uptake from soil^3^. These fine roots respond rapidly to changes in the environment. This characteristic of fine roots enables them to be used as an indicator for plant physiological status during environmental changes such as acidification or drought^4^. The response patterns of fine roots for environmental changes are usually in the form of changes in biomass^5^, morphological changes such as root diameter and specific root length^6^, and chemistry such as nitrogen concentration^4^. Fine root morphology is significant as it is critical to many functions including nutrient absorption.

The characteristics of root morphology such as root surface area, root length, and root radius determines a plant’s ability to compete for soil nutrients. Sattelmacher *et al*.^7^ reported that underground resources can be best harvested by a plant through increased root length or root surface area. In addition, N fertilisation significantly increased root tip morphology of *Pongamia pinnata* seedlings^8^. Thus, fine-root morphology shows how the plant root responds to different nutrient levels.

Nitrogen fertilisation influences root chemistry and morphology^3^. Root length and root surface area increase under intermediate levels of N fertilisation^9^. A study by Pregitzer *et al*.^10^ showed that when N is abundantly available in soil, it results in increased N concentration in roots followed by an increased rate of respiration. It has been suggested that fine root respiration facilitates carbon cycling in forests, hence, aiding the response by vegetation to global environmental changes^11, 12^. According to the published studies, root growth is triggered and maintained by root respiration, which further enhances ion absorption and transportation through the plant, especially in the xylem^13^, representing the physiological metabolic capacity of the roots^14^. Root respiration is an intricate process that encompasses both physiological and ecological mechanisms that in turn are intimately connected with root structure^15^.

Fine roots are varyingly defined as having diameters of <2 mm or <1 mm^11^, and have unique physiological functions^10, 15^. It has been shown that root respiration rates of various species and biomes are highly influenced by root N concentration, soil N availability, and temperature^16^. Guo *et al*.^3^ predicted that the higher rates of respiration can be found in first-order (i.e. finest) roots which usually have shorter a life span since their tissues contain high concentrations of nitrogen. In the present study, we combined biochar with nitrogen fertilisation to determine the effects on root growth, because although previous studies have showed positive effects of biochar on root morphology^17^, there are currently no detailed studies on the physiological and chemical changes in fine roots, or on the effect of biochar and N fertilization combined.

Nurseries use biochar to improve soil properties, especially fertility^18^. Biochar has physicochemical properties highly beneficial to plants, such as high porosity, high capacity for cation exchange which enhances nutrient retention, increased presence of beneficial microorganisms, and high water retention capacity^19^. A further study describes the benefits brought about by biochar application such as soil fertility improvement, enhanced plant growth, increased soil carbon sequestration, and reduced gas emissions^20^.

Studies have found a significant increase in root tip number (64%) and root biomass (47%) in (Larix gmelinii)^21^, increased root length in Asian rice (*Oryza sativa*)^22^, and increased lateral root length in edible asparagus, within the layer of applied biochar^17^. Therefore, it is logical to conclude that the presence of biochar leads to abundant root growth and significant positive changes in root behaviour. Van Zwieten *et al*.^23^ showed that the application of biochar on its own will only slightly increase plant growth, but it has also been shown that the combined application of biochar and nitrogen fertiliser increases plant growth, biomass, and yield^24^. Chemical fertilisers, zeolite, wood vinegar, and organic fertilisers are added to wood charcoal to increase its beneficial effects in tea plants, citrus, and vegetables^25^.

Studies have also shown promising effects on growth and plant production by combining biochar with mineral fertilisers^23, 26^. In the current study, we examined the root traits of *Acer mono* across five root orders. Specifically targeted root traits included root morphology (total root length, average root diameter, and specific root length), chemistry (tissue N concentration and C:N ratio), and physiology. We aimed to address two questions: how fine root orders vary with biochar and nitrogen addition, and how the physiological and metabolic functions vary with biochar and nitrogen addition across five root orders.

## Results

### Effect of biochar and N applications and their interaction on root morphology

**Figure 1:**
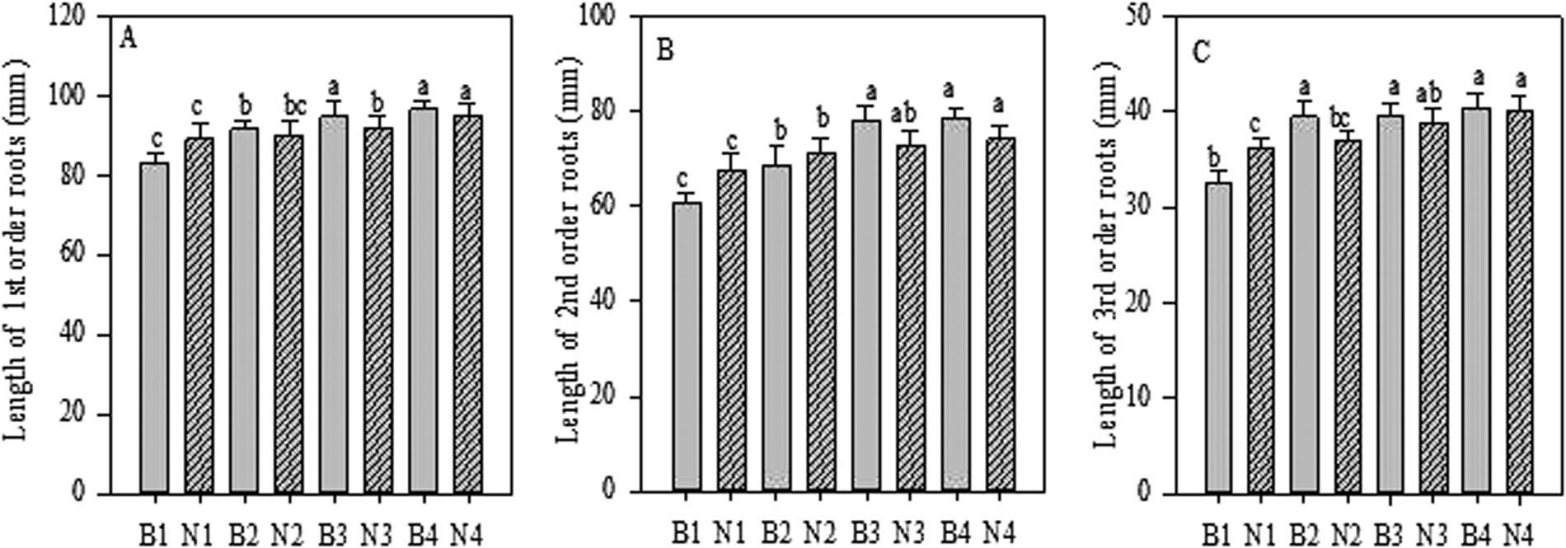
Root Length of first-third order roots after Biochar and Nitrogen addition. B stands for Biochar levels (B1-B4); N stands for Nitrogen levels (N1-N4). Different letter show the level of significance. Error bars represent the standard error of the mean.

**Figure 2:**
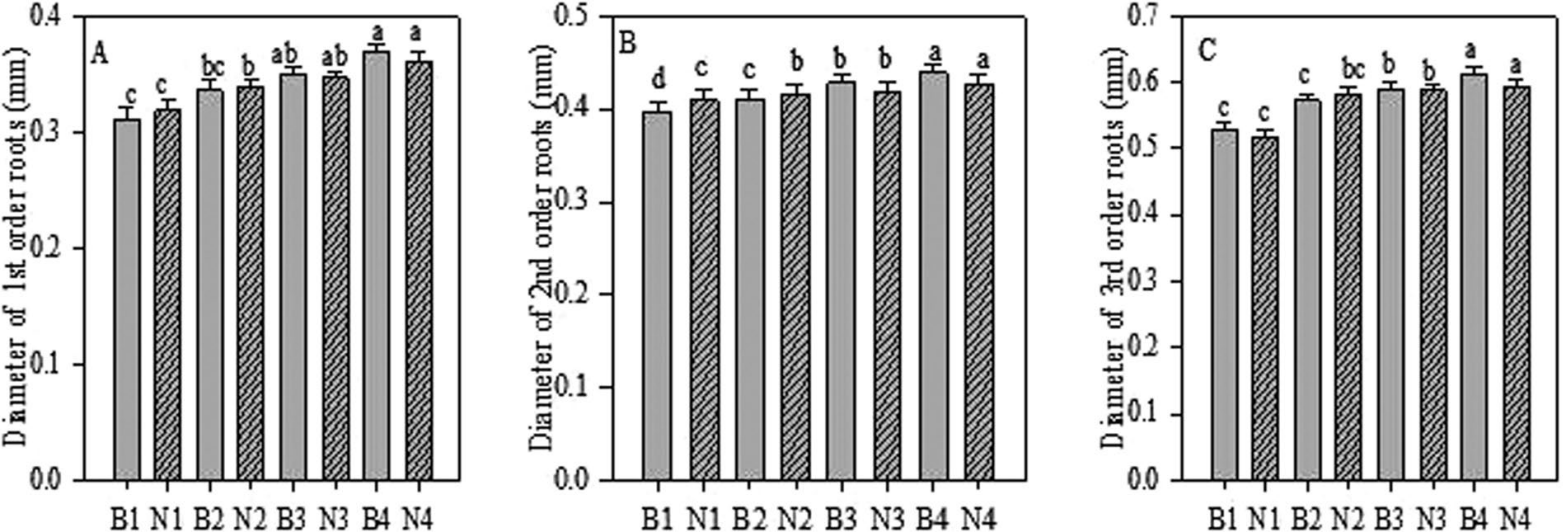
Root Diameter of first-third order roots after Biochar and Nitrogen addition. B stands for Biochar levels (B1-B4); N stands for Nitrogen levels (N1-N4). Different letter show the level of significance. Error bars represent the standard error of the mean.

**Figure 3:**
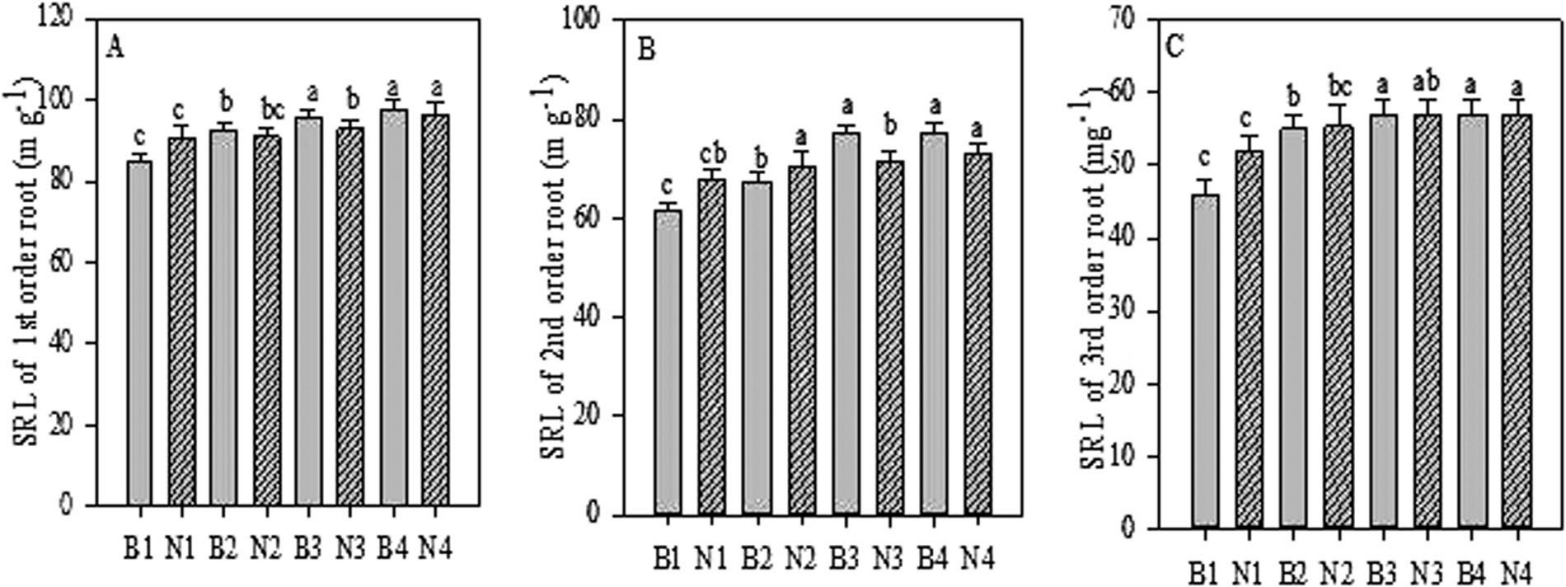
SRL of first-fifth order roots after Biochar and Nitrogen addition. B stands for Biochar levels (B1-B4); N stands for Nitrogen levels (N1-N4); SRL stand for specific root length. Different letter show the level of significance. Error bars represent the standard error of the mean.

Biochar application significantly (*p* < 0.05) increased total root length (TRL), average root diameter (ARD), and specific root length (SRL) values (Figs 1a–c, 2a–c and 3a–c), of first, second, and third root orders compared with untreated seedlings (control). The values of these three parameters were the highest with 20 g biochar per seedling. Similarly, N fertilisation significantly (*p* < 0.05) increased the root morphological index values (i.e. TRL, ARD, and SRL) of first, second, and third root orders, and the values of these parameters were highest in the seedling treated with 6 g N fertilisation (Figs 1a–c, 2a–c and 3a–c). Furthermore, biochar and N alone had no significant effect on fourth- and fifth-order roots.

We also observed a significant (*p* < 0.05) positive effect of biochar and N for TRL and SRL of first- and second-order roots, but found that interaction significant for ARD of first, second, and also third root orders. The TRL and SRL values of the first two orders, as well as the ARD values of first- to third-order roots, were highest in seedlings treated with 20 g biochar and 6 g N combined (Tables S1–S3). We observed no significant (*p* < 0.05) effect on TRL and SRL values of third, fourth, and fifth orders, nor for ARD values of fourth- and fifth-root orders (Tables S1–S3).

### Effect of biochar and N applications and their interaction on root chemistry

**Figure 4:**
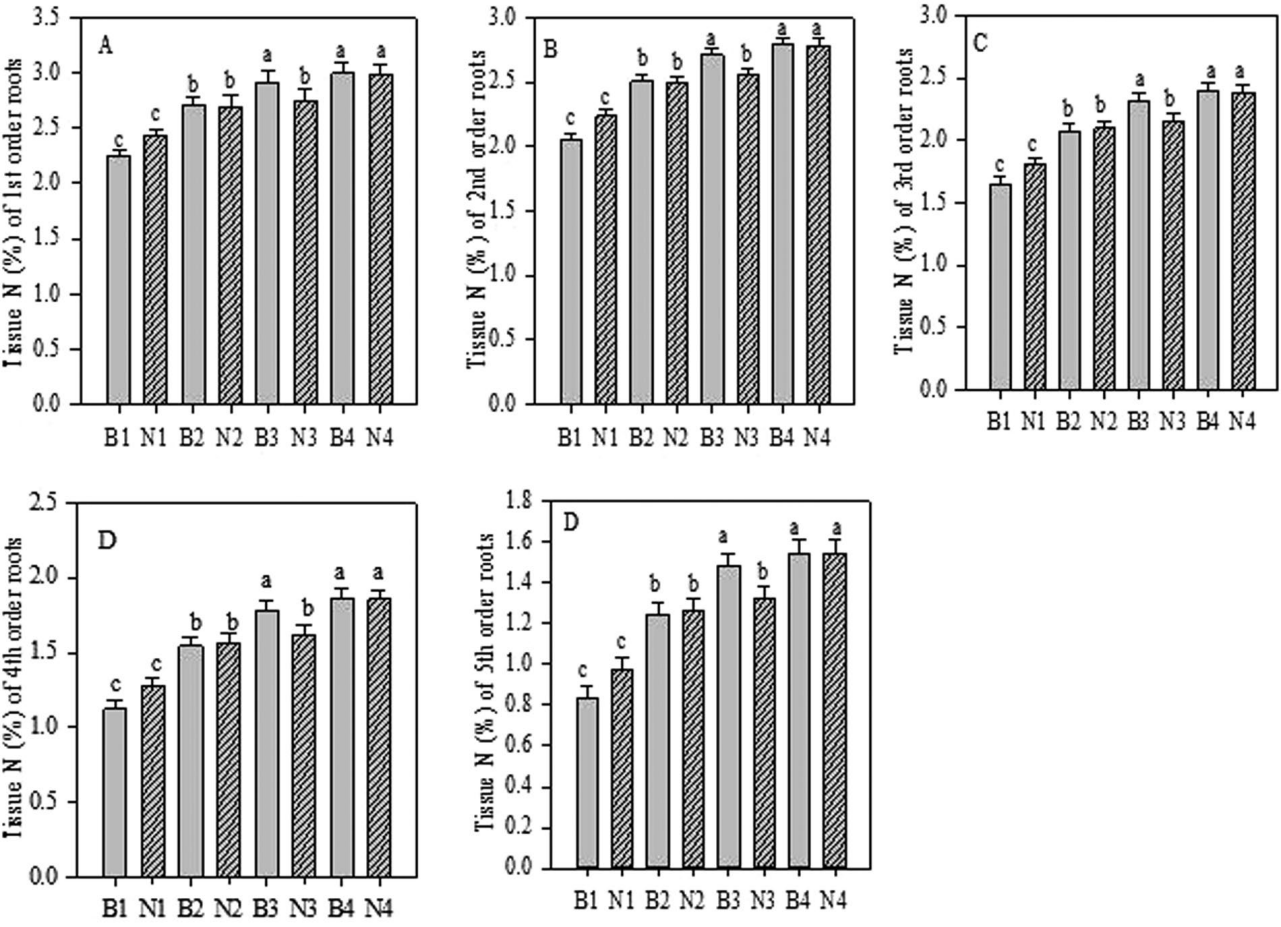
Root tissue N concentration of first-fifth order roots after Biochar and Nitrogen addition. B stands for Biochar levels (B1-B4); N stands for Nitrogen levels (N1-N4). Different letter show the level of significance. Error bars represent the standard error of the mean.

**Figure 5:**
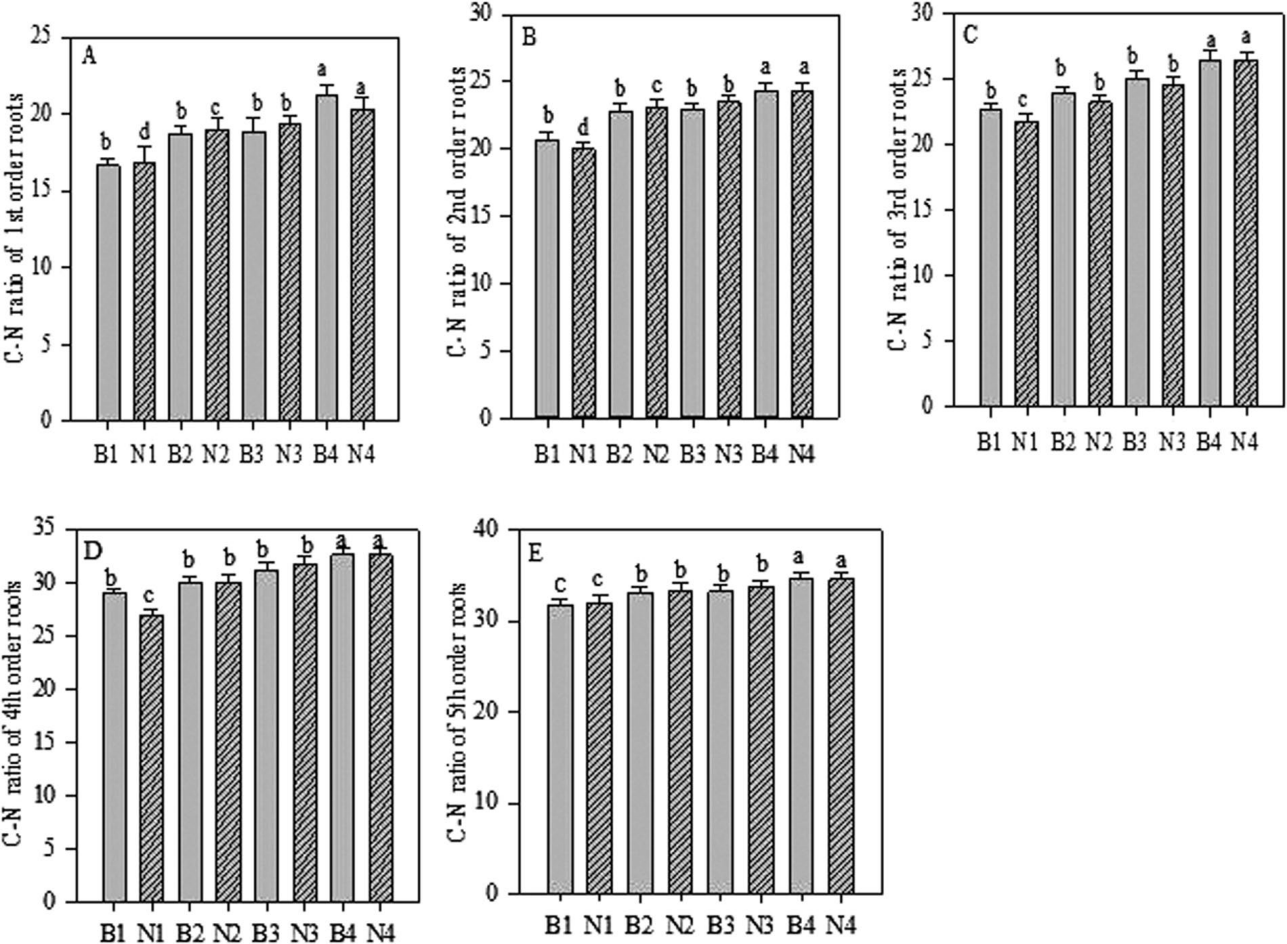
Root C- N ratio of first-fifth order roots after Biochar and Nitrogen addition. B stands for Biochar levels (B1-B4); N stands for Nitrogen levels (N1-N4). Different letter show the level of significance. Error bars represent the standard error of the mean.

Fine roots of the first five root orders treated with biochar showed significantly (*p* < 0.05) increased root-tissue N concentration and C:N ratios compared to those of control (Figs 4a–e and 5a–e). In all five root orders, the values were the highestin seedlings treated with 20 g biochar each. Similarly, N fertilisation significantly (*p* < 0.05) increased root-tissue N concentration and C:N ratio in the fine roots of the first five root orders. In all five root orders, the values were the highest in seedlings treated with 6 g N fertiliser (Figs 4a–e and 5a–e). In combination, the highest increase in root-tissue N concentration and C:N ratio in the first five root orders of the seedlings were observed when treated with 20 g biochar and 6 g N per seedling (Tables S4 and S5).

### Effect of biochar and N applications and their interaction on root respiration

**Figure 6:**
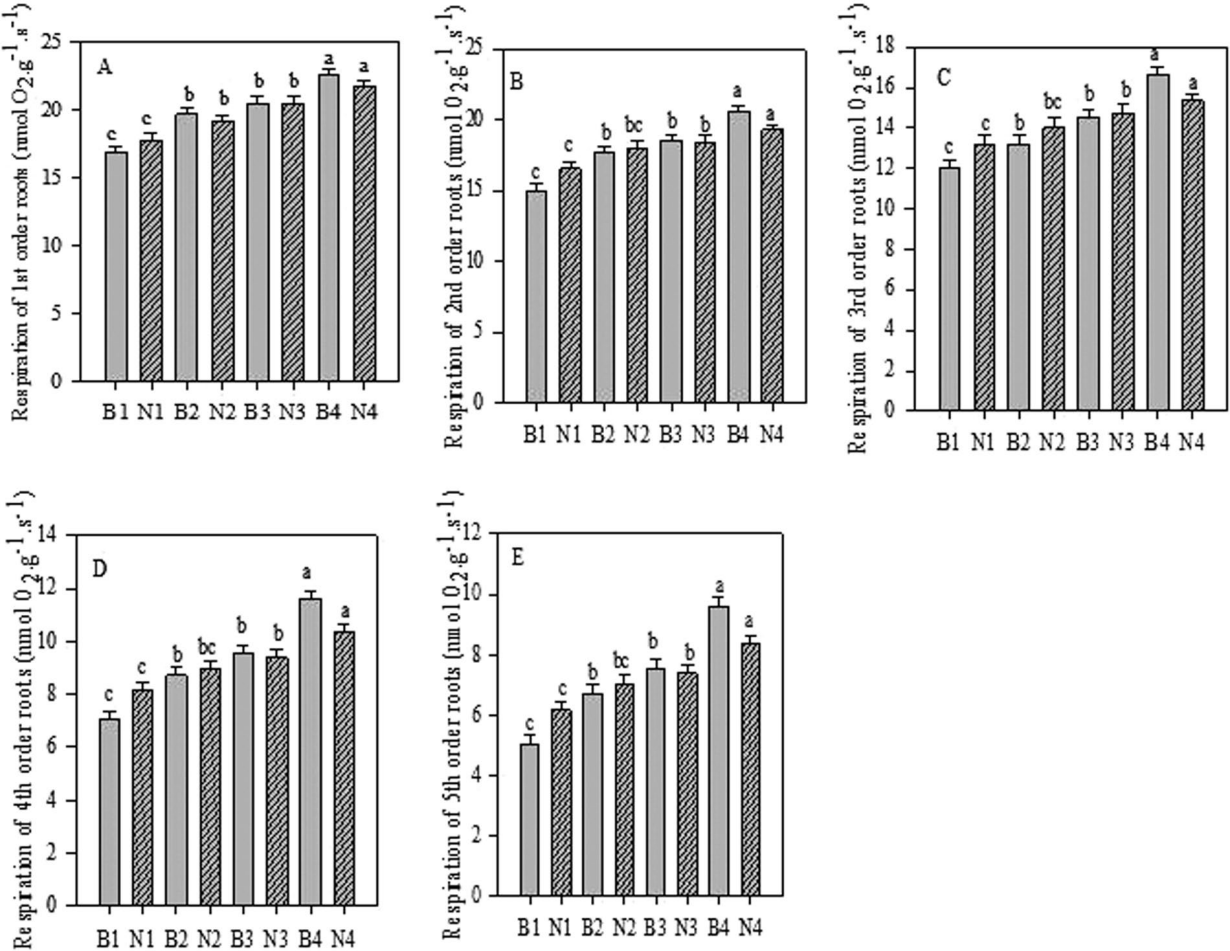
Root respiration of first-fifth order roots after Biochar and Nitrogen addition. B stands for Biochar levels (B1-B4); N stands for Nitrogen levels (N1-N4). Different letter show the level of significance. Error bars represent the standard error of the mean.

Seedlings treated with biochar significantly (*p* < 0.05) increased root respiration for first- to fifth-order roots (Fig. 6a–e). Respiration rate increased from first to fifth order with increasing biochar levels, and the highest rates were recorded in seedlings treated with 20 g biochar each. Similarly, N levels significantly (*p* < 0.05) increased root respiration rates in each order roots, and the highest values were recorded in seedlings treated with 6 g N (Fig. 6a–e). We also observed a significant (*p* < 0.05) interaction effect of biochar and N on root respiration in each order, with the highest values being recorded in seedlings treated with 20 g biochar and 6 g N (Table S6).

**Figure 7:**
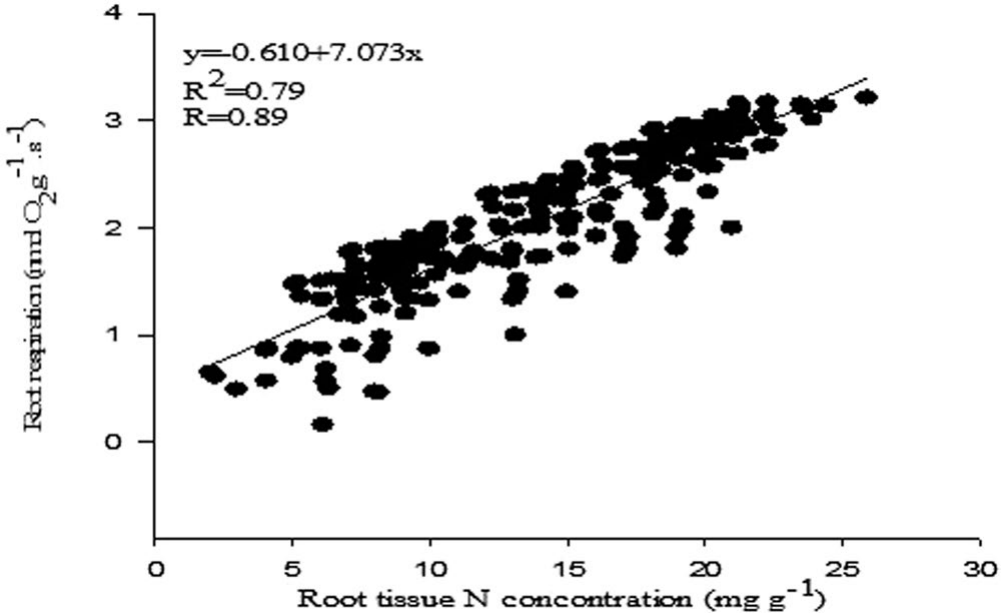
Relationship of root respiration rate and root N concentration in *Acer mono*. Data for each of three replicates, 16 treatments, and five root orders are included in the plots.

When analysed, the correlation between root respiration and tissue N concentration showed a close relationship between fine root respiration rate and root N concentration where *R* ^2^ was 0.7922 and *p* values were less than 0.001 (Fig. 7).

## Discussion

### Effect of biochar and N applications and their interaction on root morphology

Many studies have reported that biochar increases plant crop growth and yield^24^ in natural woodland as well as in cultivated fields^27, 28^. Noguera *et al*.^22^ stated that biochar application increased not only root growth but also shoot-to-root biomass ratio. Guo *et al*.^29^ reported that biochar has long been known to increase root volume, length, and surface area of *Poncirus trifoliata* (L.) Raf. seedlings in Gannan acidic red soil. Our results also showed that biochar application improved root morphology (i.e. TRL, ARD, and SRL) of *A. mono* seedlings (Figs 1a–c, 2a–c and 3a–c).

The current study findings are in agreement with the results of Yang *et al*.^30^, who concluded that during the seedling stage in a sugarcane cultivar, applying biochar improves root morphology. When bark charcoal is applied, they found that the root amount number (or biomass) significantly increased along with the increase in maize arbuscular mycorrhizal (AM) fungi colonisation rate. Ishii and Kadoya^31^ further reported that when charcoal is applied, there is an increase in root growth. Fine root morphological changes are associated with changes in soil water retention^32^ and evaporation^33^, and when these enhancements in the soil’s physical and chemical properties occur, they have a profound effect on root growth. Contrary to our results however, Deenik *et al*.^34^ found a decrease in plant growth in similar conditions following biochar application.

Biochar added to the soil forms organomineral complexes, so increasing the nutrient status and availability of the soil; it also improves microbial community composition, boosts systemic plant defences, and reduces soil pathogen load, which subsequently improve the health of plant with minimum harm to the environment^35^. Overall, these results demonstrate the potential of biochar application for improving plant growth efficiency.

When nitrogen is supplied at a plant’s seedling stage, it results in rapid growth, and when introduced after this period of rapid growth, plants obtain the ability to absorb and fix N at a higher rate. Williams and Haynes^36^ noted that the requirement for nitrogen is limited during the seedling stage but it increases hugely during subsequent growth. In most instances, the amount of nitrogen in the soil is a limiting factor on the plant’s growth, hence the need for fertiliser remains in order to support and sustain maximum growth. In the present study, there is a close relationship between nitrogen concentrations and root growth.

This relationship has been widely reported for annual crops^37^ and perennial plants^38^. Longer root lengths and greater root surface areas are obtained with medium N fertiliser concentrations, compared with either no N fertiliser or higher concentrations of fertiliser^9^. Fine root elongation significantly increases with N application^39, 40^. By contrast, N fertilisation tends to decrease fine root proliferation^41^. The above studies also conclude that the fine root response to N fertilisation is affected by soil fertility and other soil chemical conditions. Furthermore, the scale of the study area has an impact on the pattern of changes in fine root elongation. For instance, when microsites are provided with N fertilisation, the proliferation of fine roots results^42^.

In the current study, the plants in the N-fertilised plots showed higher root length and root diameter of fine roots compared to the control plots. These values were significantly higher in the first three order roots (Figs 1a–c and 2a–c). These results are in line with the pattern of N fertilisation enhancing root length and root diameter demonstrated in a previous study^9^. Similar results were obtained by Noguchi *et al*.^43^, showing an increased rate of fine root elongation in N-fertilised plots compared to control plots. In our study, fine root morphology showed a clear relationship with nitrogen addition, in that the SRL was higher in the first three orders of root compared with control plants (Fig. 3a–c). This builds on the findings of a previous study that showed that the SRL of fine roots varies with soil N concentration^39^.

When N fertilisers are used, the fine root SRL usually decreases^6^. Specifically, a study by Wang *et al*.^44^ reported that the SRL of first- and second-order roots of *Pinus tabuliformis* are decreased by N fertilisation. Ironically, under heterogeneous soil nutrient conditions, root proliferation usually happens in nutrient-rich patches^10^. Furthermore, biochar and nitrogen together significantly affected overall parameters of fine root morphology of first- and second-order roots (Tables S1–S3). Surface root length values have an important role as an index to measure the cost and benefit of fine roots to the plant, as it is assumed that the root length is proportional to the acquisition of resources, whereas the root mass is proportional to growth and maintenance^45^.

Biochar and nitrogen therefore significantly increase root proliferation of first- and second-order roots. Furthermore, SRL is positively linked with N uptake and root respiration^46^. The results of the current study showing increased fine root SRL may be because *A. mono* plants responded rapidly to the additions, so increasing the soil resource acquisition from N-rich patches. As mentioned in the literature^47^, SRL may be affected by physical properties of the soil such as porosity and bulk density. We observed that the soil’s physical and chemical characteristics did indeed affect the morphological responses of fine roots to N fertilisation.

### Effect of biochar and N applications and their interaction on root chemistry

Biochar significantly increases uptake of N in plants, as reported by Zwieten *et al*.^23^. According to a study by Chan *et al*.^48^, when treated with biochar, radish plants (*Raphanus raphanistrum* subsp. *sativus*) show a higher N uptake. This increase in uptake of N indicates the potential of biochar to improve fertiliser use efficiency in soil. Many studies^49–52^ have shown the benefits of biochar application for promoting plant development, i.e. increased above-ground biomass, higher overall growth, improved soil water-retention capacity, higher net assimilation rates, increased total content of N, P, K, Mg, Cu, and Zn leading to an increase in fine root proliferation through enhanced SRL and reduced root tissue density, and increased yield through improving the physical and biochemical properties of cultivated soils.

Our results revealed that biochar significantly increased root-tissue N concentration and C:N ratio in each order of root (Figs 4a–e and 5a–e). These increases in root tissue parameters demonstrate a positive interaction between biochar particles and roots. Salim^53^ found a significant increase in N concentration in common wheat root and leaf tissues when using biochar. Ironically, peanut (*Arachis hypogaea* L.) hull biochar showed an increase in soil N concentration with no effect on maize tissue N, when applied at 11.2 and 22.4 mg/ha^49^. A study on geographical conditions focusing on loamy sand in Georgia, US, showed no increase in soil N or tissue N when using pine (*Pinus* spp.) woodchip biochar^54^. According to Prendergast-Miller *et al*.^55^, while wheat root length is increased and root N uptake is decreased when biochar is used, there is no effect on plant biomass and plant N content.

Many studies^10, 56^ have shown that root N concentration decreases with increasing root order (Fig. 7a–e). Similarly, C:N decreases with increasing root order^56^. The C:N ratio increases as root order increases, and within each root order is highest with N treatment^10^ (Fig. 5a–e). Different species show differing responses to nitrogen fertilisation in terms of tissue N concentration. Pregitzer *et al*.^10^ reported an increase of N concentration in roots after N fertilisation in the first three root orders, but only in three out of nine temperate deciduous tree species. Likewise, there is a strong response shown by all five fine root orders toward N fertilisation, which is closely related to low soil N availability, and low baseline root-tissue N concentrations^57^.

The results of our study corroborated those of Sun *et al*.^58^, in that N fertilisation significantly increased N concentration in first- to fifth-order roots (Fig. 4a–e). The conflicting results of Pregitzer *et al*.^10^ couldbe explained through a possible relationship between baseline N concentration and root order responses to fertilisation. The lowest concentrations of soil N treatments did not alter the fine-root N concentrationsignificantly^59^, whereas the higher N treatments caused a significant increase in the total N, in fine root biomass, and in total plant biomass in deciduous treespecies^60^. Interestingly, in our study, we also found significant positive effects of biochar and N across all five root orders (Table S4). *Pinustaeda* fine roots have been shown to increase at elevated CO_2_, demonstrating the linear relationship described by the carbon-nutrient balance hypothesis^61, 62^.

### Effect of biochar and N applications and their interaction on root respiration

In the current study, the roots of different branch orders were seen to exhibit distinct differences in their rates of respiration. Specifically, the relationship between root respiration and root branch order seems to be inversely proportional, i.e. first-order roots show the highest root respiration rate compared to all fourother root branch orders (Fig. 6a–e). Biochar has been reported to influence microbial composition and activity, which may in turn affect mineralisation/immobilisation processes in the soil^2^. A study by Zak *et al*.^63^ inferred that there is a significant increase in microbial biomass C around the rhizosphere and in the bulk soil region when plants are grown under elevated CO_2_ conditions.

In conditions where soil nutrients are abundant, morphology^64^, tissue N concentration and soil respiration^65^, and fine root biomass^66^ are all affected. The impact on root respiration in such conditions, however, varies according to different parameters including measurement methods, root diameter, soil nutrient condition, and time of fertilisation^16^. Generally, the addition of N fertilisers increases the rate of root respiration^59^. For instance, when seedlings of *P. Taeda* were fertilised, they showed increased root respiration^67^. Drake *et al*.^16^ reported that when free-air CO_2_ enrichment and N fertilisation are combined, a reduction of about 40% in fine root respiration results.

The current study showed that the rate of root respiration increased in the first five root orders and was highest with combined N fertilizer and biochar application compared to control (Table S4). The reason for increased root respiration with N fertilisation may be an increase in root-tissue N concentration^11^. A positive correlation exists between root-tissue N and the energy for protein turnover and ion exchange (i.e. maintenance respiration)^68^, whereas increased availability of nitrogen is related to enhanced metabolic activity per unit root mass^11^. As nitrogen concentration in root tissues increases when N is introduced as fertiliser, this ultimately results in increased root respiration. A further mechanism that helps to increase root respiration through N fertilisation is an increase in the efficiency of root N uptake^66^.

The process of respiration is connected with nitrogen concentration in tissues of different organs such as fine roots, leaves, and stems^69^. The current study showed that there was a high correlation between root-tissue N concentration and root respiration (Fig. 7), as concluded in a previous study^11^.

A study has shown that physiological activity is reflected in the root respiration rate^70^. In the current study, the increased respiration in first-order roots seems to be consistent with higher metabolic activity when compared with higher order roots. There is also a relationship between physiological functions, and branch age and order^71^; and nitrate uptake and root respiration both decline rapidly as root age increases^72^. Furthermore, the variation in root functions is manifested in tissue N concentrations. The positive correlation of respiration rate with N concentration in our study is consistent with the theory that maintenance respiration depends on an adequate tissue-protein concentration^57^.

## Conclusion

Our results demonstrate that biochar and nitrogen strongly interact to influence *A. mono* fine-root morphology, chemistry, and respiration. The strongest effects on root traits and physiology are seen when both sufficient organic matter and N are available. Our study found a positive influence of biochar and nitrogen on root morphology in first- to third-order roots, and a significant positive effect on root chemistry across the five root orders. Moreover, the combination of biochar and N increased the tissue nitrogen concentration, which in turn has a linear relationship with root respiration, and hence increases the root respiration of the individual roots. Overall, our study reveals that a combination of biochar and nitrogen had a profound effect on root traits of *A. mono*. However, literature is scarce on the influence of biochar and N, especially on root traits, chemistry, and respiration, and therefore further studies are needed to explore the mechanisms and potential applications of these relationships.

### Study site and soil collection

This experiment was conducted at the Maoershan experimental station for seedling growing (127°′‒127°′E, 45°23′‒45°26′ N, 390 m above sea level), which is located in the temperate forest region in Heilongjiang Province of Northeast China. The station has cold environmental conditions with continental monsoon climate where 2.8 °C remains to be the average air temperature on yearly basis whereas in January and July, the average temperatures are −19.6 °C and 20.9 °C, respectively. The annual average humidity, annual precipitation, and annual evaporation are 70%, 723.8 mm, and 1094 mm, respectively. And the soil is mostly dark-brown earth, which belongs to Boric Luvisols in the classification system of Chinese Soil Taxonomy^73^; the frost-free period usually lasts from 120 to 140 d.

Soil samples were collected from the experiment field at 0–20 cm soil depth and were thoroughly mixed to make a representative composite soil sample that had a total N, P, and K content of 3.98 g kg^−1^, 820.8 mg kg^−1^, and 14 g kg^−1^, respectively, and an available N, P, and K content of 4, 7.23, and 176 mg kg^−1^.

### Plant materials and fertilization treatments

Four-year-old *A. mono* Maxim L. seedlings of uniform size were selected in a field nursery for study. Planting spacing was 20 cm × 30 cm. Each seedling was treated with one of four levels of biochar B1, B2, B3, B4 (0, 10, 15, or 20 g) and one of four levels of N1, N2, N3, N4 (0, 2, 4, or 6 g). Ten plants were included in each treatment, and each treatment was replicated three times (total n = 480). We applied nitrogen in two split doses during May and July, and biochar was applied once as a basal dose in May. Standard cultural practices were performed during the experiment (i.e., weeding, hoeing, irrigation, etc.), in order to produce healthy seedlings.

### Root morphology measurement

**Figure 8:**
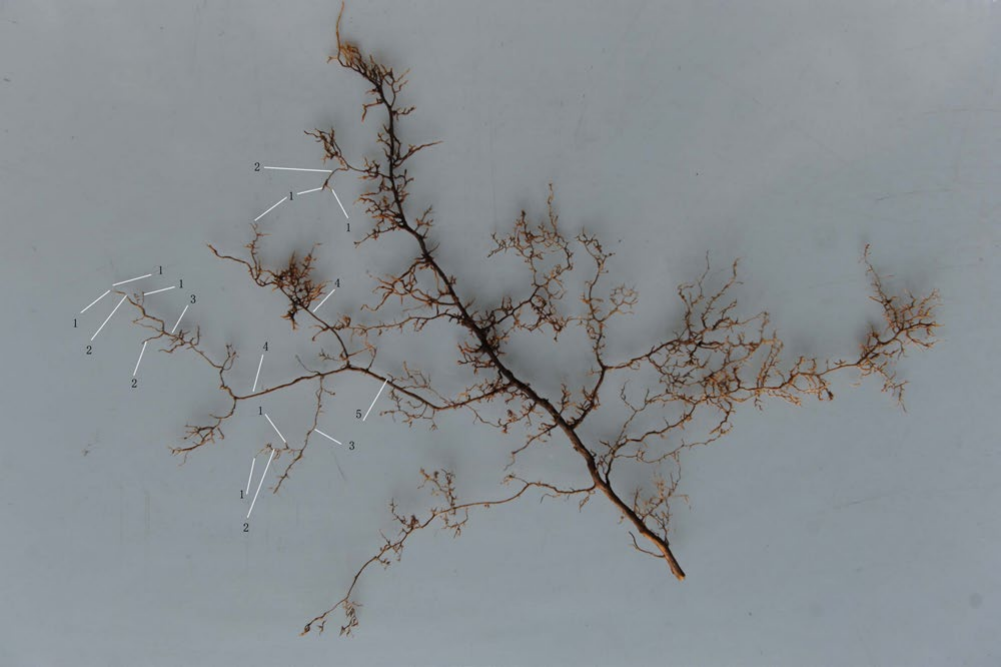
*Acer mono* root orders from first to fifth orders.

Root samples were carefully taken in each treatment group during harvesting, using the procedure described by Guo *et al*.^15^. After harvesting, the root samples were shifted to the laboratory in an ice box within four hours. The individual samples were cleaned with de-ionized water to remove residual soil particles and stored in a refrigerator. As per Pregitzer *et al*.^10^ method, distal branch order as the first order, the root samples of each treatment were divided into different branch orders (Fig. 8). The samples separated as mentioned above were scanned with Expression 10000XL 1.0 scanner (dpi = 400; Epson Telford, Ltd., Telford, UK) following which the images were analyzed with the help of WinRHIZO (Pro2004b) software (Instruments Regent Co., Ville de Québec, QC, Canada) in order to measure the Average Root Diameter (ARD) and Total Root Length (TRL). Finally, the roots were oven dried to constant mass at 65 °C, in order to determine dry mass, and SRL was calculated as the TRL from each root order divided by the corresponding dry mass^74^.

### Root respiration and root tissue N concentration

In September 2016, fine roots were collected at random locations within each plot with the help of a specially constructed 20 × 20 cm rectangular soil corer that is equipped with sharpened edges root samples were hand-mixed and packed in the plastic bags and then transported to the laboratory for measuring root respiration. Every day, sampling was done twice at different time intervals (6:00 and 17:00 hours) whereas within four hours of collection, the samples were measured for root respiration. After washing with deionized water gently, each composite sample was used.

After cutting the living roots from branch nodes, all the five root branch orders are sorted with root tips designated as first-order roots as per the procedure by Pregitzer *et al*.^10^. 0.5 g of root samples were allowed to equilibrate in the water at respective measurement temperatures for 30 min which is then followed by monitoring O_2_ consumption for 30 more minutes using gas-phase O_2_ electrodes (Model LD 2/2, Hansatech Instruments Ltd, King’s Lynn, UK) connected to constant-temperature circulating water baths^11^. Post completion of respiration measurements, the root samples were oven-dried at 75 °C for 24 h and weighed. Root respiration was calculated as nmol O_2_ g^−1^ s^−1^ (dry weight). Once the drying and weighing is done, every treatment’s root samples and root order, its total N and C:N concentration were measured with the help of a Macro Elemental Analyzer (vario MACRO, Elementar Co., Germany).

### Statistical analysis

The experiment was conducted in a randomised complete block design, with split-plot arrangements so that the effects of biochar, nitrogen fertiliser, and their interaction on seedlings, root morphology, root physiology, and chemistry could be tested. The data collected was analysed using two-way analysis of variance (ANOVA) with the software package SPSS 21.0 (IBM, Guildford, UK). LSD test was also performed for the experiments to compare treatments with one another. The correlations between total root N concentration and root respiration were performed using Sigma Plot 12.5 (Systat Software, San Jose, CA). Significance levels were set at 0.05.

## Electronic supplementary material

Supporting information
