# Supplementary material for: Influence of biochar and nitrogen on fine root morphology, physiology, and chemistry of Acer mono
Source: Sci Rep. 2017 Jul 14;7:5367. doi: 10.1038/s41598-017-05721-2 (PMC5511266; doi:10.1038/s41598-017-05721-2)
Supplement: Supplementary file 1 — Supporting information [file 41598_2017_5721_MOESM1_ESM.pdf]

Supporting information

Influence of biochar and nitrogen on fine root morphology, physiology, and chemistry of *Acer mono*

Muhammad Razaq[1], Salahuddin[1,2], Hai-long Shen[1], Hassan Sher[3], Peng Zhang [1*]

Table S1. Root Length of first-fifth order roots after Biochar and Nitrogen combine addition. B stands for Biochar levels (B1-B4); N stands for Nitrogen levels (N1-N4). Different letter show the level of significance. ± represent the standard error of the mean.

Treatment	RL order 1st	RL order 2nd	RL order 3rd	RL order 4th	RL order 5th
B1N1	78.3±3.2g	50.0±2.7e	NS	NS	NS
B1N2	83.3±3.0fg	60.3±2d	NS	NS	NS
B1N3	82.6±4.9 f	65.0±3.1c	NS	NS	NS
B1N4	88.6±5.5ef	67.6±3.1bc	NS	NS	NS
B2N1	87.0±4.2ef	67.3±3.1bc	NS	NS	NS
B2N2	89.6±4.4de	68.6±3.7bc	NS	NS	NS
B2N3	93.0±4.2cd	68.3±3.3bc	NS	NS	NS
B2N4	97.0±5.6bc	69.6±3.6b	NS	NS	NS
B3N1	93.0±2.2cd	76.6±3.9a	NS	NS	NS
B3N2	97.0±5bc	79.0±3.9a	NS	NS	NS
B3N3	95.6±4.9bc	77.6±2.3a	NS	NS	NS
B3N4	93.0±5.5cd	78.6±3.9a	NS	NS	NS
B4N1	98.3±4.2a	76.6±3.9a	NS	NS	NS
B4N2	89.6±5.4de	77.3±4.1a	NS	NS	NS
B4N3	96.6±4.2bc	79.0±4.1a	NS	NS	NS
B4N4	102±5.6a	80.3±4.7a	NS	NS	NS

Table S2. Root diameter of first-fifth order roots after Biochar and Nitrogen combine addition. B stands for Biochar levels (B1-B4); N stands for Nitrogen levels (N1-N4). Different letter show the level of significance. ± represent the standard error of the mean.

	RD order 1st	RD order 2nd	RD order 3rd	RD order 4th	RD order 5th
B1N1	0.274±0.01g	0.364±0.02l	0.556±0.02h	NS	NS
B1N2	0.298±0.01fg	0.386±0.02kl	0.580±0.02fgh	NS	NS
B1N3	0.314±0.02def	0.401±0.02jk	0.577±0.02efg	NS	NS
B1N4	0.320±0.01bcde	0.407±0.03ijk	0.590±0.04e	NS	NS
B2N1	0.323±0.02cde	0.403±0.02fghi	0.565±0.02ef	NS	NS
B2N2	0.336±0.01cde	0.405±0.03hij	0.583±0.02de	NS	NS
B2N3	0.343±0.04cde	0.408±0.02hij	0.579±0.02ef	NS	NS
B2N4	0.343±0.05cde	0.412±0.04ghij	0.607±0.03efgh	NS	NS
B3N1	0.348±0.04def	0.427±0.03def	0.573±0.02cd	NS	NS
B3N2	0.351±0.03bcd	0.428±0.05cde	0.559±0.03ef	NS	NS
B3N3	0.352±0.02bcd	0.424±0.04defg	0.600±0.03cd	NS	NS
B3N4	0.352±0.02bcd	0.433±0.04bcd	0.619±0.04bc	NS	NS
B4N1	0.365±0.04bcde	0.418±0.04efgh	0.581±0.03cd	NS	NS
B4N2	0.366±0.03bcd	0.439±0.05bc	0.570±0.04ef	NS	NS
B4N3	0.382±0.05ab	0.442±0.07b	0.603±0.06ab	NS	NS
B4N4	0.395±0.06a	0.456±0.07a	0.626±0.07a	NS	NS

Table S3. Specific root length (SRL) of first-fifth order roots after Biochar and Nitrogen combine addition. B stands for Biochar levels (B1-B4); N stands for Nitrogen levels (N1-N4). Different letter show the level of significance. ± represent the standard error of the mean.

	SRL order 1st	SRL order 2nd	SRL order 3rd	SRL order 4th	SRL order 5th
B1N1	81.6±2g	54.3±2e	NS	NS	NS
B1N2	84.3±3fg	60.3±2d	NS	NS	NS
B1N3	83.6±2fg	64±3cd	NS	NS	NS
B1N4	89.6±4de	66.6±3bc	NS	NS	NS
B2N1	88±4ef	66.3±3bc	NS	NS	NS
B2N2	90.6±4de	67.6±3bc	NS	NS	NS
B2N3	94±5cd	67.3±3bc	NS	NS	NS
B2N4	98±5bc	68.6±3b	NS	NS	NS
B3N1	94±4cd	75.6±4a	NS	NS	NS
B3N2	98±5bc	78±4a	NS	NS	NS
B3N3	96.6±5bc	76.6±4a	NS	NS	NS
B3N4	94±4cd	77.6±4a	NS	NS	NS
B4N1	99±5b	75.6±4a	NS	NS	NS
B4N2	90.6±5de	76.3±4a	NS	NS	NS
B4N3	97.6±5bc	78±4a	NS	NS	NS
B4N4	104±7a	79.3±4a	NS	NS	NS

Table S4. Root tissue N concentration of first-fifth order roots after Biochar and Nitrogen combine addition. B stands for Biochar levels (B1-B4); N stands for Nitrogen levels (N1-N4). Different letter show the level of significance. ± represent the standard error of the mean.

Treatment	TNC order 1st	TNC order 2nd	TNC order 3rd	TNC order 4th	TNC order 5th
B1N1	1.90±0.1f	1.70±0.1f	1.30±0.05d	0.83±0.03d	0.58±0.03d
B1N2	1.96±0.3f	1.76±0.2f	1.36±0.05d	0.94±0.03d	0.54±0.02d
B1N3	2.20±0.2ef	2.04±0.2ef	1.64±0.05d	1.11±0.03d	0.91±0.03c
B1N4	2.82±0.3bcd	2.42±0.3bcd	2.22±0.05abc	1.19±0.03abcd	1.00±0.04bc
B2N1	2.31±0.4e	2.31±0.3e	2.22±0.05abc	1.05±0.04d	0.75±0.02d
B2N2	2.79±0.4bcd	2.59±0.2bcd	2.19±0.06abc	1.66±0.04abc	1.36±0.03abc
B2N3	2.84±0.4bcd	2.64±0.3bcd	2.24±0.06abc	1.71±0.05abc	1.41±0.04abc
B2N4	2.87±0.4bcd	2.67±0.3abcd	2.27±0.06abc	1.74±0.05abc	1.44± 0.04abc
B3N1	2.82±0.4bcd	2.62±0.2bcd	2.22±0.06abc	1.69±0.04abc	1.39±0.04abc
B3N2	2.96±0.4bcd	2.76±0.2abc	2.36±0.06abc	1.83±0.05abc	1.43±0.05abc
B3N3	2.77±0.4cd	2.57±0.3cd	2.17±0.06abc	1.84±0.05abc	1.44±0.04abc
B3N4	3.09±0.5ab	2.79±0.2ab	2.49±0.06ab	1.86±0.04abc	1.58±0.05ab
B4N1	2.63±0.4a	2.83±0.3d	2.03±0.06c	1.77±0.04abc	1.60±0.05ab
B4N2	3.04±0.5abc	2.84±0.3abc	2.44±0.06ab	1.95±0.04ab	1.66±0.05a
B4N3	3.15±0.5a	2.95±0.2a	2.55±0.06a	2.01±0.05a	1.68±0.06a
B4N4	3.16±0.5a	2.96±0.3a	2.56±0.06a	2.02±0.05a	1.71±0.06a

Table S5. C: N ratio of first-fifth order roots after Biochar and Nitrogen combine addition. B stands for Biochar levels (B1-B4); N stands for Nitrogen levels (N1-N4). Different letter show the level of significance. ± represent the standard error of the mean.

	N: C order 1st	N: C order 2nd	N: C order 3rd	N: C order 4th	N: C order 5th
B1N1	16. ±0.3h	21±0.4h	22.6±0.4h	28.7±0.7h	30.8±0.5g
B1N2	17.5±0.2ef	21.3±0.4gh	23.8±0.4g	29.9±0.7g	32±0.5f
B1N3	19±0.2de	21.7±0.4fg	24.2±0.5g	30.3±0.7fg	32.3±0.5ef
B1N4	19±0.2cd	22.6±0.4de	24.4±0.5fg	30.5±0.7fg	32.6±0.6ef
B2N1	17.90±0.2efg	22.3±0.5de	24.5±0.6fg	30.6±0.7fg	32.7±0.6ef
B2N2	18.5±0.2ef	22±0.5ef	24.7±0.7efg	30.8±0.9efg	32.9±0.6def
B2N3	18.5±0.3ef	22.6±0.5de	24.7±0.6efg	30.8±0.9efg	32.9±0.7def
B2N4	19.9±0.4c	22.4±0.5de	24.7±0.7efg	30.8±0.9efg	32.9±0.7def
B3N1	17.6±0.3gh	23.4±0.5cd	25.3±0.7def	31.4±0.9def	33.4±0.7cde
B3N2	18.2±0.3fg	23.6±0.5cd	25.7±0.7cde	31.8±0.8cde	33.9±0.7cd
B3N3	19.7±0.4cd	23.8±0.6cd	26±0.7bcd	32.1±0.9bcd	34.2±0.7bc
B3N4	19.8±0.3c	23.5±0.5cd	26±0.7bcd	32.1±0.9bcd	34.2±0.7bc
B4N1	18.3±0.4efg	24±0.6bc	26.1±0.7bcd	32.2±0.8bcd	34.3±0.7bc
B4N2	20.7±0.3b	24.3±0.bc	26.4±0.9bc	32.5±0.9bc	34.6±0.9bc
B4N3	20.2±0.5bc	24.8±0.6b	26.9±0.9b	33±0.9b	35.1±0.9ab
B4N4	21.7±0.6a	25.8±0.7a	27.9±0.9a	34±0.9a	35.9±0.9a

Table S6. Root respiration of first-fifth order roots after Biochar and Nitrogen combine addition. B stands for Biochar levels (B1-B4); N stands for Nitrogen levels (N1-N4);

(Mean ± standard error). Different letter show the level of significance. ± represent the standard error of the mean.

Treatment	Rep order 1st	Rep order 2nd	Rep order 3rd	Rep order 4th	Rep order 5th
B1N1	15±0.5g	13.2±0.6f	9.3±0.4f	4.3±0.3f	2.4±0.2g
B1N2	18.4±0.5f	16.4±0.6e	12.4±0.4e	7.4±0.3e	5.5±0.3f
B1N3	18.5±0.5f	16.5±0.6e	12.5±0.4e	7.5±0.3e	5.5±0.2f
B1N4	19.4±0.5ef	17.4±0.6de	13.4±0.3de	8.4±0.4de	6.4±0.2ef
B2N1	19.6±0.5ef	17.6±0.6de	13.6±0.4de	8.6±0.4de	6.6±0.3ef
B2N2	19.6±0.5def	17.6±0.6de	13.6±0.4de	8.6±0.4de	6.6±0.3ef
B2N3	19.7±0.5def	17.7±0.7de	13.7±0.3de	8.7±0.4de	6.7±0.2def
B2N4	19.9±0.5cdef	17.9±0.7cde	13.9±0.4cde	8.9±0.4cde	6.9±0.3cdef
B3N1	20±0.6cdef	18±0.7cde	14±0.4cde	9±0.4cde	7±0.3cdef
B3N2	20.2± 0.6cdef	18.2±0.7cde	14.2±0.4cde	9.2±0.4cde	7.2±0.3cdef
B3N3	20.9±0.6bcde	18.9±0.7bcd	14.9±0.4bcd	9.9±0.3bcd	7.9±0.4bcde
B3N4	20.9±0.6bcde	18.9±0.7bcd	14.9±0.4bcd	9.9± 0.3bcd	7.9±0.4bcde
B4N1	21.6±0.6bcd	19.6±0.7bc	15.6±0.4bc	10.6±0.3bc	8.6±0.4bcd
B4N2	21.7±0.6bc	19.7±0.7bc	15.7±0.5bc	10.7±0.4bc	8.7±0.4bc
B4N3	22.3±0.6b	20.3±0.7b	16.3±0.5b	11.3±0.4b	9.3±0.4b
B4N4	24.6±0.6a	20.6±0.7a	18.6± 0.5a	13.3± 0.4a	11.6±0.4a
